# Integrative GWAS and transcriptomic analyses identify candidate genes associated with oil content and fatty acid composition in safflower

**DOI:** 10.1186/s12870-025-08024-1

**Published:** 2026-01-29

**Authors:** Somayeh Sardouei-Nasab, Fatemeh Mahdavinasab, Reza Haghi, Ghasem Mohammadi-Nejad, Azam Nikbakht-Dehkordi, Zahra Nemati

**Affiliations:** 1 Research and Technology Institute of Plant Production, Afzalipour Research Institute, Shahid Bahonar University of Kerman, Kerman, Iran; 2 Department of Plant Production and Genetics, College of Agriculture, Shahid Bahonar University of Kerman, Kerman, Iran; 3 Leibniz Institute of Plant Genetics and Crop Plant Research (IPK), Gatersleben, Germany

**Keywords:** Fatty acid composition, Lipid metabolism, QRT-PCR, Safflower, Oil content, Transcriptome

## Abstract

**Background:**

This study investigates the genetic basis of oil content, fatty acid (FA) composition, and seed yield in safflower (*Carthamus tinctorius* L.) to support breeding efforts aimed at improving oil quality and yield. A genome-wide association study (GWAS) was conducted on 262 safflower accessions using 4,545 SNP markers to identify genetic loci associated with oil-related traits. Additionally, RNA-seq was performed to analyze gene expression differences between high- and low-oleic safflower varieties.

**Results:**

GWAS identified 64 significant genetic loci, with chromosome 10 showing a high concentration of markers linked to key oil traits, including oleic acid, linoleic acid, vaccenic acid, oil content, and oil yield. Transcriptome analysis revealed 206 genes involved in lipid metabolism, including those related to FA synthesis and oil accumulation. Several transcription factors were also identified as putative regulators of these processes. Real-time quantitative reverse transcription PCR (qRT-PCR) confirmed the expression levels of five selected differentially expressed genes, chosen based on statistical significance and functional relevance to lipid metabolism.

**Conclusions:**

The integration of GWAS and transcriptome analyses provided valuable insights into the genetic and molecular mechanisms underlying oil production in safflower. These findings can be applied to develop safflower varieties with enhanced oil content and quality, benefiting both agricultural and industrial applications.

**Supplementary Information:**

The online version contains supplementary material available at 10.1186/s12870-025-08024-1.

## Introduction

 Safflower (*Carthamus tinctorius* L.) is an annual, diploid (2*n* = 2x = 24), self-pollinated oilseed crop [1]. It is a valuable source of high-quality edible oil, particularly adapted for cultivation in moisture-limited environments [2]. In recent years, safflower has gained global prominence as a modern industrial oilseed crop due to its excellent agronomic performance, high oleic and linoleic acid content, and the associated health benefits and oxidative stability of oleic acid, making it ideal for culinary use without the need for partial hydrogenation [3, 4]. Similarly, safflower oil is prized for its stability and high quality in food applications. This is attributed to its low peroxide value, high content of total tocopherols, abundant polyunsaturated fatty acids and omega-6 acids, and low saturated fat content [5].

High-oleic safflower oil contains 71–79% oleic acid, which is comparable to high-oleic canola oil (71–79%) and high-oleic soybean oil (72–75%) but lower than high-oleic sunflower oil (80–93%). Despite not having the highest oleic acid content, high-oleic safflower oil offers a distinct advantage due to its very low alpha-linolenic acid (ALA, C18:3) content, typically < 1% and often as low as ~ 0.04% [6]. In contrast, high-oleic canola oil contains ~ 2% ALA, high-oleic soybean oil contains ~ 3–7% ALA, and high-oleic sunflower oil contains ~ 0.1–1% ALA [7]. Because ALA is highly unsaturated and prone to oxidation, its minimal presence in safflower oil enhances oxidative stability, making it particularly suitable for high-heat cooking and industrial applications. This low ALA content also simplifies genetic modifications to further improve safflower oil’s stability compared to canola, soybean, and sunflower oils [8].

Additionally, safflower is recommended in areas where average annual rainfall is below 430 mm; under optimal conditions with no dry winds, it can even grow with annual rainfall as low as 300 mm annual rainfall. During summer, temperatures up to 43 °C do not cause significant problems for this crop, even under prolonged drought conditions [7].

Despite its potential, the safflower genome remained incomplete until recently. Its haploid genome size was previously estimated to be approximately 1.4 Gbp [1], although more recent sequencing-based assemblies suggest a size closer to 1.17 Gbp. Wu et al. [9] reported a chromosome-level genome assembly representing 12 pseudochromosomes, covering 1.06 Gbp (~ 90.6%) of the genome.

Genes associated with oil content and FA composition have been extensively studied in major oil crops and Arabidopsis. For example, two lipase genes (HH-026818-RA and HH-025320) were reported to participate in glycerolipid metabolism and FA degradation, leading to the breakdown of oil bodies and membrane lipids, as revealed by integrated proteome and lipidome analyses in oil crops [10]. In Arabidopsis, the expression level of the *OLE* gene was higher in high-oil seeds compared to low-oil seeds [11]. In safflower, upregulation of stearoyl-acyl carrier protein desaturase *(SAD)* and downregulation of Fatty Acid Desaturase 2 *(FAD2)* during seed development at 10 DAF may be critical for oleic acid biosynthesis, a pattern comparable to findings from high- and low-oil *Camellia oleifera* transcriptomes [12]. Other genes and enzymes known to regulate lipid metabolism in oil crops include ABHD family members such as *ABHD6*, which participate in both lipid synthesis and degradation [13]; β-ketoacyl-CoA synthases (KCS) that initiate the FA synthesis pathway during elongation [14] and function as part of the FA elongase complex under the control of the *FAE1* gene [15].

Previous GWAS in safflower have identified multiple loci associated with oil content and fatty acid composition. Ambreen et al. [16] reported 5, 3, and 5 MTAs for oil, oleic acid, and linoleic acid content, respectively, using microsatellite markers. Zhao et al. [17] identified 54 MTAs for seed oil content using SNP markers and an MLM-based GWAS approach, while Fan et al. [18] found 32 InDel loci significantly associated with FA composition (*p* < 0.01) in 605 germplasm accessions using the GLM model. By combining GWAS with RNA-Seq, we can pinpoint candidate genes that not only associate with desirable traits but also show differential expression in those samples with extreme differences in the trait of interest. Several studies have successfully applied these integrative approaches [18–20]. In the present study, we utilized a reference-based approach that combines GWAS and RNA-Seq analyses to identify candidate genes associated with oil-related traits in safflower. By integrating these approaches, we aimed to uncover both genetic loci and gene expression patterns that contribute to oil biosynthesis in safflower seeds. Our findings are expected to provide insights into the genetic and molecular mechanisms underlying oil biosynthesis, offering valuable information for future crop improvement strategies and advancing the development of safflower as a prominent oilseed crop.

## Materials and methods

### Plant materials and sample collection

For the GWAS study, we analyzed 262 safflower (*Carthamus tinctorius* L.) accessions from diverse global regions (Table S1), which were grown in a field experiment during 2016 at Shahid Bahonar University, Kerman, Iran (30.25°N, 57.10°E; 1760 m above sea level). The experimental design was an incomplete block with two replications. Seed yield was measured as yield per plant (Seed_YLD: kg/ha). Mature seeds were collected, dried, and analyzed for FA composition. Seeds were sourced from RTIPP-SBUK (Shahid-Bahonar University) and the genebank of IPK (Leibniz Institute of Plant Genetics and Crop Plant Research, Gatersleben, Germany). For RNA-seq, we selected two contrasting genotypes based on oleic acid oil content: CT71 (75.45% oleic acid) and CT217 (0.33% oleic acid). Both genotypes were cultivated in the IPK greenhouse under 16 h of light and 8 h of dark conditions at 24 °C ± 2 °C. All seed exchange and usage complied with the Nagoya Protocol and followed standard material transfer agreements.

### FA composition analysis

Seed oil concentration was determined using Soxhlet extraction with petroleum ether, following the AOCS Official Method Am 2–93 (American Oil Chemists’ Society) [21]. The FA composition was analyzed by gas chromatography (GC), including myristic acid (C14:0), palmitic acid (C16:0), stearic acid (C18:0), oleic acid (C18:1), linoleic acid (C18:2), vaccenic acid (C18:1), and eicosenoic acid (C20:1). Triacylglycerols (TAGs) were converted to fatty acid methyl esters (FAMEs) for GC analysis using INSO 14,880 standards [20]. Oil yield (Oil_YLD) was calculated by multiplying seed yield (kg/ha) by seed oil percentage [23].

We used a linear mixed model to determine adjusted genotype means. Best Linear Unbiased Estimators (BLUE) were computed from replicate data. Replication, plot, and incomplete block were treated as random effects, while safflower accessions were fixed effects. The model was represented as Y = Xβ + Zu + e, where Y denotes phenotypic data, β is the fixed effect, u is the vector of random effects, X is the design matrix for fixed effects, Z is the design matrix for random effects, and e is the random residual. Correlation analysis of seed oil-related traits was performed and visualized using the R package Performance Analytics [24].

### Genotyping-by-sequencing and SNP calling

For genotyping, a Genotyping-By-Sequencing (GBS; [25]) approach was utilized to analyze the genetic variation. The library preparation involved the use of two restriction enzymes, *Pst*I and *Msp*I, as described in the protocol by Wendler et al. [26]. Sequencing was performed on an Illumina NovaSeq 6000 device (SP reagent kit, v1.5 chemistry), generating 100-base-pair (bp) single-end reads. The quality of the raw sequencing data was initially assessed using FASTQC v.0.11.9 [27] to check for common issues such as low-quality bases and sequence duplication. Adapter sequences were trimmed, and low-quality bases were removed using CUTADAPT v.4.0 [28]. After removing the individual sample barcodes and quality filtering, our GBS data resulted in, on average, 4250 loci per individual with 98 × coverage and with 6.4% missing sites. The first chromosome-scale reference genome for safflower [9] was sequenced using the PacBio Sequel platform (Pacific Biosciences) in the ‘Anhui-1’ cultivar known for its high linoleic acid (LA) content. This reference genome demonstrated its usefulness already in the study of Karami-Moalem et al. [29] and was subsequently employed in the current study for reference-based genotyping analysis. IPYRAD v.0.9.56 [30] was employed to process the sequencing data and align it to the reference genome, allowing for the identification of single-nucleotide polymorphisms (SNPs).

SNP calling was performed using SAMtools v.1.19 [31], focusing on identifying biallelic SNPs. The filtering criteria included a minor allele frequency (MAF) of ≥ 0.01. To handle missing genotype data, BEAGLE v.4.0 [32] was used for imputation, which enhances the reliability of the genetic data by filling in missing genotypes based on a probabilistic model that considers the linkage disequilibrium patterns among SNPs.

### GWAS analysis

GWAS was performed using the rMVP package in R [33], applying GLM, MLM, and FarmCPU models. The FarmCPU model integrates multiple markers as covariates in a stepwise MLM to mitigate confounding effects. Population structure was adjusted using the first three principal components (PCs) and the kinship matrix (K) calculated by the VanRaden method [34]. Following previous GWAS studies [35–37], a fixed significance threshold of –log10(P) > 3 was applied to identify significant SNPs. To highlight the most robust associations, a stricter Bonferroni-corrected threshold at a significance level of 5% was also applied [38]. SNPs exceeding this threshold were considered highly significant. Manhattan and quantile-quantile (Q-Q) plots generated using the rMVP package.

Linkage disequilibrium (LD; r²) was calculated for all pairwise combinations of SNPs using TASSEL 5.0 T [39]. LD decay patterns were subsequently visualized by plotting r² against the physical distance between SNP pairs (bp) using the R package ggplot2 [40].

Candidate genes were examined within a ± 200 kb window surrounding each significant SNP based on the LD decay analysis. The corresponding genomic sequences were extracted from the reference genome and analyzed using *AUGUSTUS* [41] for potential open reading frames (ORFs). Functional annotation was performed using Arabidopsis-specific annotations obtained from The Arabidopsis Information Resource (TAIR; https://www.arabidopsis.org) [42]. Additional functional insights were obtained from the PANTHER classification System [43].

### Transcriptomic analysis

Developing seeds were collected 14 days post-flowering, and total RNA was isolated using the RNeasy Plant Mini Kit (QIAGEN). Three independent biological replicates were collected for each of the HO and LO cultivars. RNA quality was assessed with Qubit™ 4 Fluorometer and RNA integrity number (RIN) via microcapillary electrophoresis. Each replicate was sequenced separately. Libraries were prepared using the Illumina Stranded mRNA Prep Ligation protocol and sequenced on the Illumina NovaSeq 6000 system with 2 × 100 cycles (paired-end).

For our data analysis, the chromosome-scale reference genome of safflower [9] was used as the reference genome. Read quality was assessed with FASTQC. For quality control, each RNA sample was analyzed in technical duplicates. Trimmomatic v.0.39 [44] was used for preprocessing to remove low-quality reads and adapters. Sequencing reads ranged from 35 to 109 bp, with a predominant length of 109 bp.

Short reads were aligned to the reference genome, and raw read counts for each annotated gene were extracted using HTSeq-count with the reference annotation (.gtf) file. Only uniquely mapped reads were considered for gene-level quantification. Differentially expressed transcripts (DEGs) were identified by comparing transcript counts from high oil content samples to those with low oil content. Statistical significance was determined using the R package of edgeR [45] with thresholds set at an adjusted p-value ≤ 0.05 and |fold change| ≥ 2.

To explore the functional relevance of the DEGs, we conducted gene ontology (GO) and KEGG (Kyoto Encyclopedia of Genes and Genomes) pathway enrichment analyses. Functional enrichment was then analyzed in gProfiler (https://biit.cs.ut.ee/gprofiler/gost), using known genes of *Arabidopsis thaliana* with an adjusted p-value ≤ 0.05, owing to its well-annotated genome and comprehensive functional data. This analysis covered GO terms for cellular components, molecular functions, and biological processes, as well as KEGG pathways, providing insights into the biological functions and pathways enriched in the DETs.

###  qRT-PCR validation of selected transcripts

To validate the RNA-seq results, five differentially expressed genes (DEGs) were selected for quantitative PCR analysis. Specific primers were designed using Primer3 (v. 0.4.0) [22] (Table S2). qRT-PCR was conducted using the LightCycler 96 system (Roche, Germany), with the housekeeping gene *actin* serving as an internal control for normalization. The qRT-PCR analysis was carried out with three biological replicates for both the HO and LO cultivars. Gene expression levels between samples with low and high oil content were compared using the ΔΔCt method as described by Livak and Schmittgen [46]. ΔΔCt values were calculated as ΔCt(LO) – ΔCt(HO), with HO serving as the calibrator.

## Results

### Fatty acid composition analysis

**Fig. 1:**
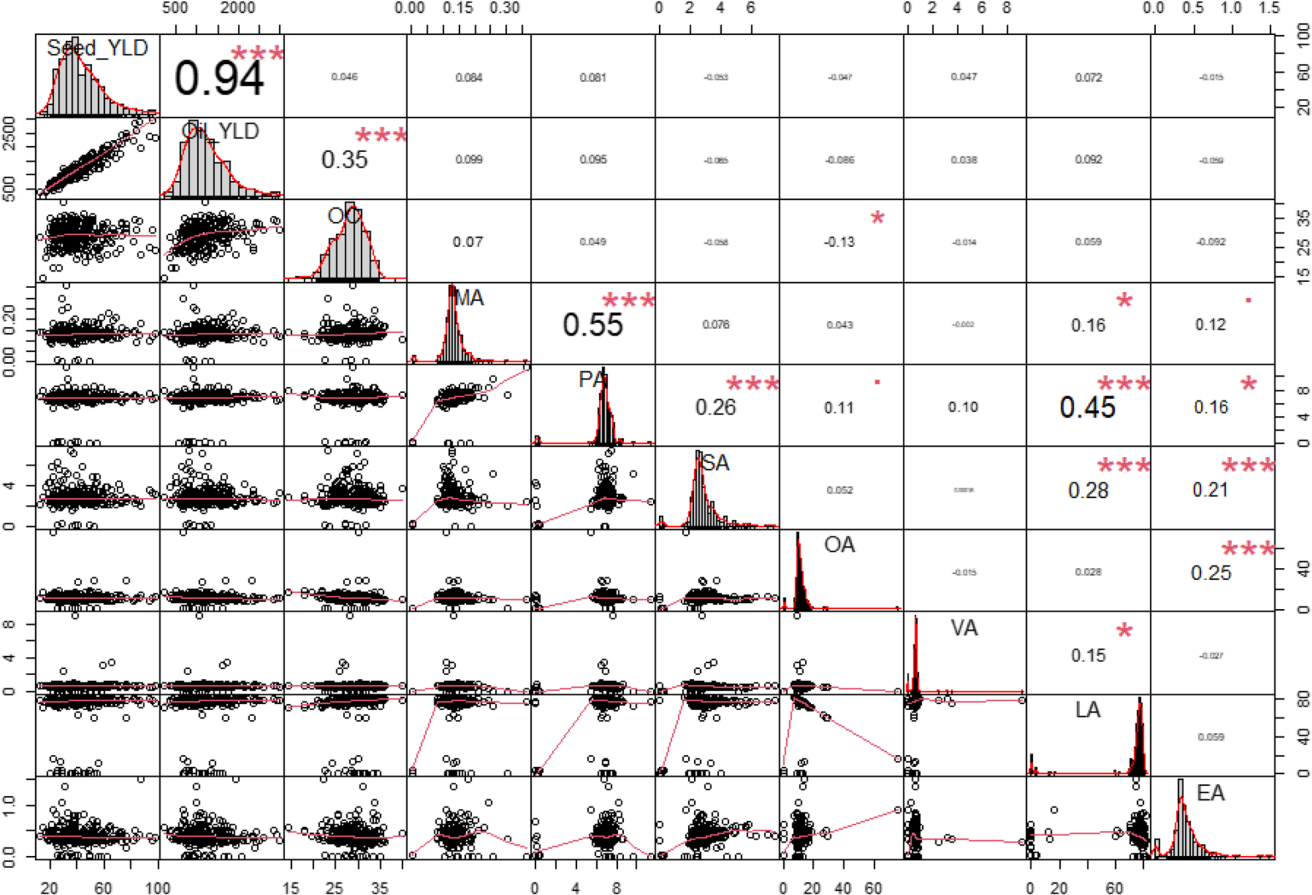
Correlation matrix of oil-related traits and seed yield in safflower seeds. The diagonal represents the distribution of each trait, while the lower diagonal displays bivariate scatter plots with fitted regression lines. The upper diagonal shows Pearson’s correlation coefficients (r) along with significance levels indicated by stars (*p < 0.05; **p < 0.01; ***p < 0.001). Significant correlations include strong positive relationships between *Seed*_*YLD* seed yield and Oil_*YLD* oil yield, as well as correlations between various FAs such as *MA* Myristic acid, *PA* Palmitic acid, and *SA* stearic acid, indicating potential shared biosynthetic pathways

The Pearson correlation analysis of oil-related traits and seed yield in safflower (Fig. 1) revealed several significant relationships. There is a very strong positive correlation between seed yield (Seed_YLD) and oil yield (Oil_YLD), with a correlation coefficient of *r* = 0.94, indicating a highly significant relationship. Oil content (OC) also exhibits a moderate positive correlation with oil yield (*r* = 0.35). Among FAs, a significant positive correlation is observed between myristic acid (MA) and palmitic acid (PA) (*r* = 0.55***). Stearic acid (SA) shows a notable positive correlation with PA (*r* = 0.26***). Additionally, oleic acid (OA) has a negative correlation with oil content (OC) but a positive correlation with eicosenoic acid (EA) (*r* = 0.25**).

### GWAS analysis

**Fig. 2:**
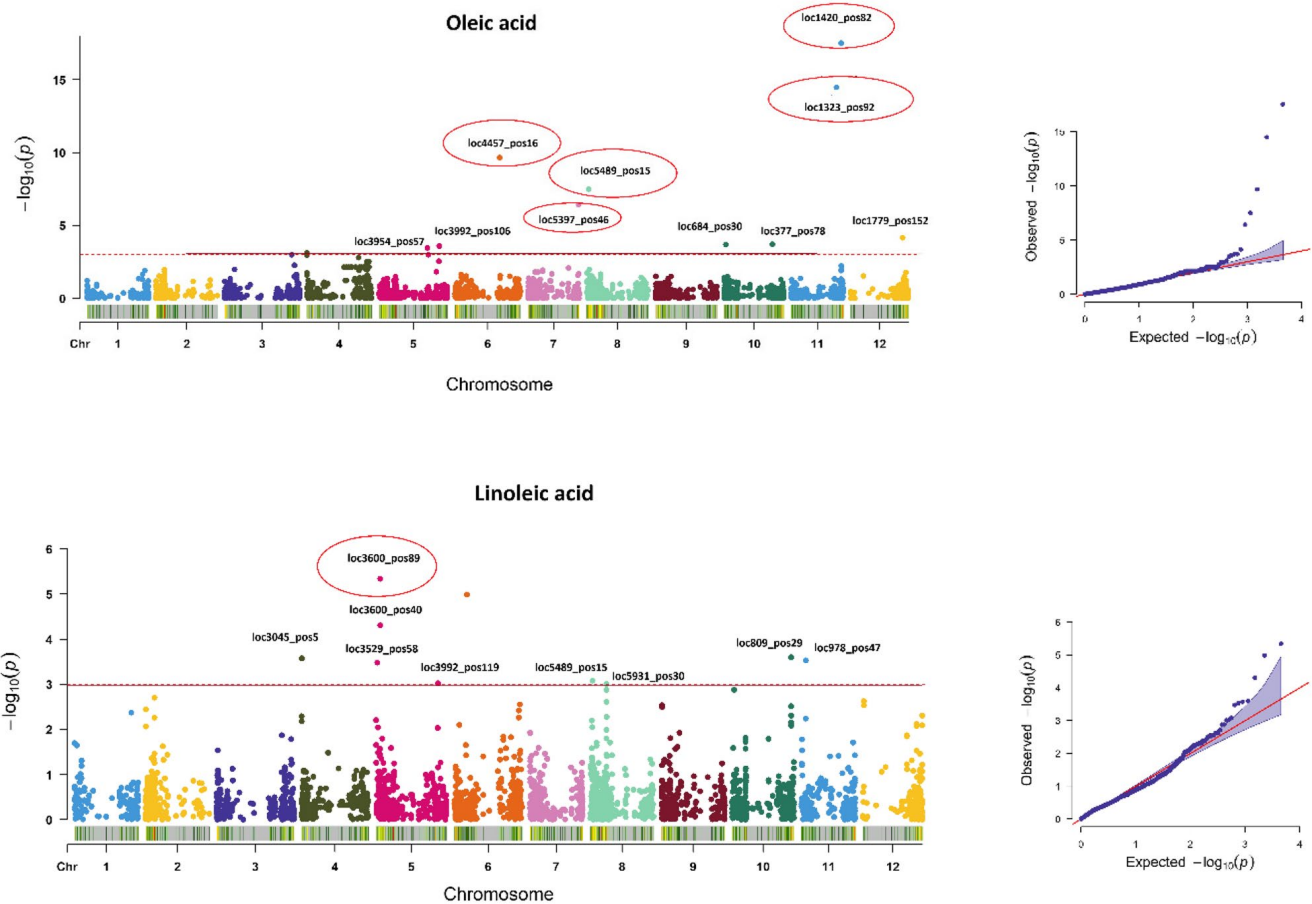
Manhattan plots for Oleic acid (*OA*) and Linoleic acid (*LA*) content. The right panels show quantile–quantile (*QQ*) plots of observed versus expected *p*-values generated using the FarmCPU method. SNPs circled in the Manhattan plots represent highly significant associations that exceeded the Bonferroni-corrected threshold (–log₁₀(*P*) ≥ 4.96)

We conducted a GWAS to identify genetic loci associated with nine oil-related traits in safflower using 4,546 SNPs mapped onto the 12 pseudochromosomes of the safflower genome assembly. Circular Manhattan plots were employed to illustrate SNPs associated with various traits by comparing three models, GLM, MLM, and FarmCPU to determine the most suitable model for each trait. False associations were evaluated using QQ plots, which led to the selection of the MLM model for seed yield (Seed_YLD) due to its p-values closely aligning with expected values along the diagonal line, while the FarmCPU model was chosen for oil-related traits (OA, LA, EA, VA, SA, PA, MA, OC, and oil yield). SNPs associated with OA and LA are presented in Fig. 2, while SNPs related to other traits are shown in Fig. S1 in the supplementary materials. GWAS analysis revealed a total of 64 significant marker-trait associations (MTAs) across the traits examined (Table 2). For OA, we detected eleven significant SNPs across chromosomes 4, 5, 6, 7, 8, 10, and 11. LA showed notable associations with ten SNPs on chromosomes 4, 5, 6, 8, 10, and 11, with most of these SNPs having negative effects on LA content, except for SNP loc3992_pos119. SNP loc5489_pos15 on chromosome 8 was associated with both OA and LA, indicating pleiotropic effects, with a positive impact on OA and a negative effect on LA. SNPs on chromosome 5, including loc3992_pos106 (OA) and loc3992_pos119 (LA), shared a common allele but had different alternate alleles. For VA, significant SNPs were identified across various chromosomes, with SNP loc681_pos19 on chromosome 10 located near SNP loc684_pos30 associated with OA. EA was associated with eight significant SNPs spread across different chromosomes. Octadecenoic acid (OCn) was linked to nine SNPs within two genomic regions: one on chromosome 10 (7,560,290 to 7,904,336 bp) and another on chromosome 6 (88,676,969 to 88,681,230 bp). Significant SNPs for Seed_YLD were also identified, including loc640_pos68 on chromosome 10 (positive effect) and loc6269_pos21 on chromosome 8 (negative effect). To ensure a more stringent threshold, a second cutoff was applied using a Bonferroni correction for multiple testing, resulting in a Bonferroni-corrected threshold of –log₁₀(P) ≥ 4.96 for SNP markers. Using this threshold, nine SNPs—loc4457_pos16, loc5397_pos46, loc5489_pos15, loc1323_pos92, loc1420_pos82 (chromosomes 6, 7, 8, and 11, OA), loc3600_pos40 (chromosome 5, LA), loc2238_pos60, loc6652_pos3 (chromosomes 2 and 9, EA), and loc4053_pos7 (chromosome 5, PA), were identified as highly significant (α/n) and are bolded in Table 1.

**Table 1:** Marker–trait association (MTA) results for seed yield and oil-related traits in safflower, as identified using MLM and FarmCPU models

Trait	SNP	Chr.	Position (pb)	*P*-Value	REF	ALT	Effect
Oleic acid	loc377_pos78	10	2,428,985	0.000205	G	A	−2.52824
	loc684_pos30	10	68,694,192	0.000191	A	T	2.457316
	**loc1323_pos92**	**11**	**64,686,625**	**3.38E-15**	**C**	**T**	**−48.7501**
	**loc1420_pos82**	**11**	**71,419,608**	**3.03E-18**	**C**	**T**	**6.354586**
	loc1779_pos152	12	73,821,555	7.11E-05	G	A	−2.59433
	loc3043_pos21	4	285,380	0.000775	A	T	1.377717
	loc3954_pos57	5	68,712,471	0.000353	G	T	2.830642
	loc3992_pos106	5	85,633,330	0.000256	G	A	1.131378
	**loc4457_pos16**	**6**	**64,027,079**	**2.16E-10**	**C**	**T**	**−5.98746**
	**loc5397_pos46**	**7**	**72,120,436**	**3.82E-07**	**T**	**G**,** A**	**−4.11512**
	**loc5489_pos15**	**8**	**3,135,949**	**3.30E-08**	**G**	**T**	**6.048404**
Linoleic acid	loc809_pos29	10	81,097,476	0.000252	A	G	−7.8824
	loc978_pos47	11	5,957,938	0.000297	A	C	−16.3746
	loc3045_pos5	4	419,332	0.000266	A	C	−18.5044
	loc3529_pos58	5	1,926,449	0.000333	G	T	−11.8322
	**loc3600_pos40**	**5**	**5,794,010**	**4.96E-05**	**A**	**G**	**−15.4107**
	loc3600_pos89	5	5,794,059	4.59E-06	C	T	−18.7967
	loc3992_pos119	5	85,633,343	0.000959	C	G	6.131435
	loc4300_pos68	6	17,667,736	1.03E-05	A	C	−22.9808
	loc5489_pos15	8	3,135,949	0.000829	G	T	−16.4498
	loc5931_pos30	8	22,232,077	0.000985	A	G	−7.01238
Eicosenoic acid	loc1279_pos28	11	62,256,498	0.000373	T	C	0.053562
	loc1902_pos77	12	79,524,189	0.000412	G	T	−0.14036
	**loc2238_pos60**	**2**	**12,551,047**	**7.15E-06**	**C**	**A**	**0.174686**
	loc2240_pos51	2	12,569,291	8.52E-05	C	T	0.153861
	loc2252_pos67	2	13,264,673	0.000164	C	G	0.111715
	loc4709_pos124	6	91,030,417	0.000286	A	G	0.188347
	loc6268_pos5	8	85,041,896	1.25E-05	C	G	0.214827
	**loc6652_pos3**	**9**	**19,733,203**	**7.58E-06**	**G**	**A**	**0.215822**
Vaccenic acid	loc101_pos15	1	9,191,255	0.000967	A	G	0.465743
	loc681_pos19	10	68,503,965	1.42E-05	T	G	0.946136
	loc1297_pos55	11	63,106,910	2.57E-05	A	C	0.549456
	loc2205_pos7	2	11,281,827	0.000338	T	C	−0.23362
	loc2433_pos83	2	76,800,022	0.000551	T	G	0.6259
	loc3394_pos7	4	85,020,433	0.000759	T	C	0.247864
	loc3394_pos25	4	85,020,451	0.000886	G	C	0.242965
	loc4282_pos62	6	13,868,478	0.000494	G	A	0.576096
	loc4302_pos96	6	17,837,999	1.77E-05	G	A	0.778207
	loc6168_pos23	8	59,689,422	0.000451	C	A	0.587174
	loc6555_pos21	9	13,500,494	0.000163	T	G	0.631017
Stearic acid	loc2797_pos5	3	76,123,350	3.95E-05	A	G	1.030282
	loc4853_pos65	7	5,429,603	2.43E-05	G	A	−0.37379
	loc6136_pos34	8	50,467,679	0.000456	A	C	0.844471
Palmitic acid	loc289_pos38	1	78247463	4.78E-05	G	A	−1.3251
	loc289_pos61	1	78247486	4.78E-05	C	T	−1.3251
	loc289_pos92	1	78247517	4.78E-05	T	C	−1.3251
	loc3501_pos51	5	266570	9.06E-05	T	C	0.702881
	**loc4053_pos7**	**5**	**97014529**	**1.06E-05**	**A**	**G**	**−1.37894**
Myristic acid	loc2952_pos7	3	1.01E+08	0.000634	A	G	0.022236
	loc3899_pos8	5	28864600	0.000386	G	A	0.026718
Oil content	loc431_pos20	10	7560290	0.000924	G	A	1.192158
	loc433_pos56	10	7619178	0.000263	T	C	1.305305
	loc434_pos29	10	7625780	0.000421	C	A	1.281256
	loc434_pos32	10	7625783	0.000263	C	A	1.305305
	loc435_pos41	10	7904312	0.000158	T	G	1.375091
	loc435_pos65	10	7904336	0.000158	A	C	1.375091
	loc4655_pos52	6	88676969	0.000651	G	A	2.010736
	loc4656_pos25	6	88681185	0.000805	C	G	2.174343
	loc4656_pos70	6	88681230	0.000805	T	C	2.174343
Oil-Yield	loc640_pos68	10	50301754	0.000672	A	C	0.063829
	loc6269_pos21	8	85061398	0.00088	C	T	−0.02226
Seed_Yield	loc3691_pos7	5	10362796	0.000724	G	C	8.573482
	loc3692_pos20	5	10377614	0.000912	G	A	6.609163
	loc5031_pos54	7	16063922	0.000529	C	T	10.75946

*REF* Reference allele, *ALT* Alternative allele,

Bolded SNPs represent highly significant associations exceeding the Bonferroni-corrected threshold (–log₁₀(P) ≥ 4.96)

**Fig. 3:**
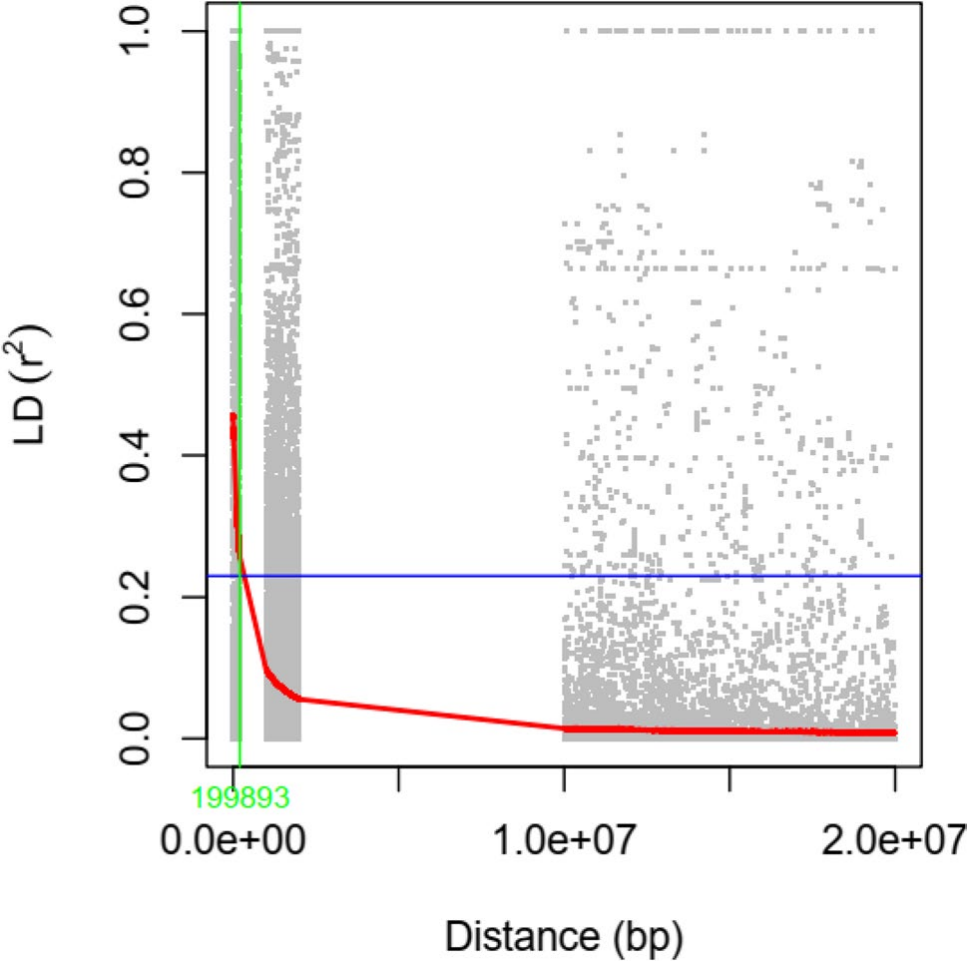
Linkage disequilibrium (LD) decay plot in safflower based on pairwise $\:{r}^{2}$values between SNP markers. The red curve represents the fitted LD decay trend, while the blue horizontal line indicates the critical $\:{r}^{2}$threshold (0.2). The green vertical line marks the half-decay distance (~ 200 kb)

In addition to identifying significant SNPs, candidate genes were examined within a ± 200 kb window of these MTAs, based on LD decay analysis, as the half-decay distance was estimated at approximately 200 kb (Fig. 3). We identified 1,915 candidate genes in the safflower genome (Table S3). These genes are involved in critical processes such as abscisic acid (ABA)-activated signaling, FA biosynthesis, and lipid metabolism. These genes function in roles such as pyruvate dehydrogenase (acetyl-transferring) activity, lysophospholipid acyltransferase activity, enoyl-[acyl-carrier-protein] reductase (NADH) activity, GTPase activity, acetyl-CoA carboxylase activity, 3-oxoacyl-[acyl-carrier-protein] synthase activity, hydrolase/lipase activity, as well as lipid catabolism and lipid binding. Genes associated with the ABA signaling pathway, such as those linked to SNP loc4282_pos62 on chromosome 6 and SNP loc2205_pos7 on chromosome 2, were found to correlate with vaccenic acid. Similarly, genes associated with linoleic acid, oleic acid, eicosenoic acid, and palmitic acid were identified near their respective SNP loci. Additionally, 73 identified genes were transcription factors (TFs) from various families, including AGAMOUS-like MADS-box, APETALA2-like, and MYB, which may play roles in FA biosynthesis and metabolism.

### Transcriptome analysis

We analyzed a total of 6 RNA-seq samples from two extreme genotypes of safflower with high-oleic oil content (HO) and low-oleic oil content (LO), each with three biological replicates, resulting in 41.50 Gb of data and 202,500,012 raw reads.

We utilized Hisat2 to map the RNA-seq reads to the reference genome, achieving approximately 85% of clean reads mapped, with 82% of these uniquely mapped. After applying a filter to the count matrix (minimum count > 10 in all samples), we identified 49,889 transcripts, which included 18,397 annotated genes (prefix CtAH) and 31,492 novel transcripts (prefix MSTRG). Our analysis revealed 6,521 DEGs with significant expression changes (p-value < 0.05 and fold change > 2), with expression levels ranging from − 18 to 19 times (Tables S4, S5). Of these DEGs, 1,939 were matched to annotated genes (CtAH prefix), and 4,582 were novel transcripts (MSTRG prefix). Among the DEGs, 2,956 were down-regulated and 3,565 were up-regulated in the high-oleic (HO) genotypes.

**Fig. 4:**
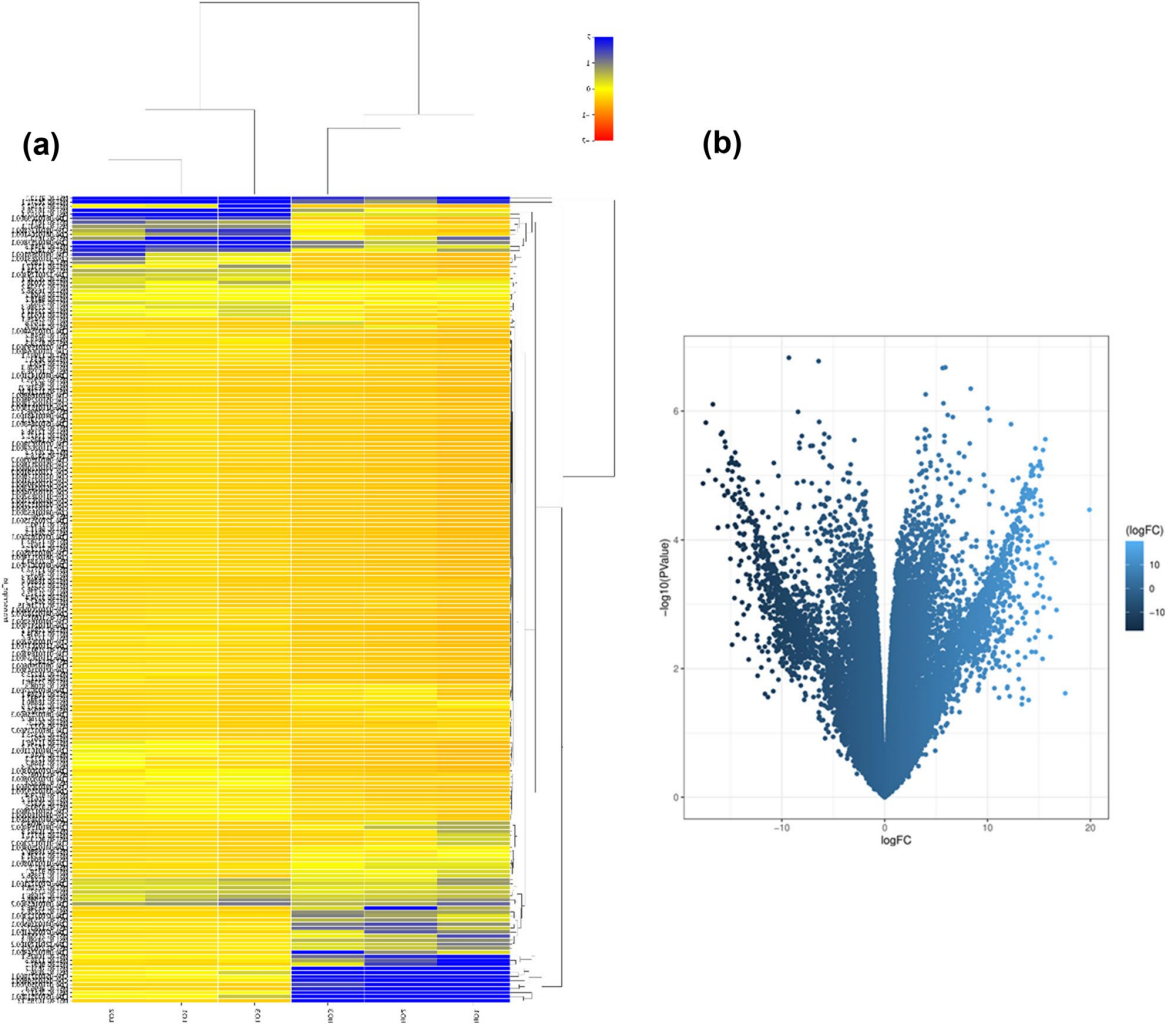
Volcano plot and heatmap of differentially expressed genes (DEGs) in safflower genotypes with high- and low-oleic acid content. **(a)** The heatmap displays expression profiles of 206 DEGs involved in oil biosynthesis across safflower genotypes. Rows represent genes, and columns represent samples. The color scale (blue to yellow) indicates expression levels. Two distinct clusters reflect expression differences between high- and low-oleic acid content genotypes, revealing the genetic factors affecting oil composition. (**b)** The volcano plot displays the Log2 fold change (logFC) against the -Log10 p-value for genes. The color gradient indicates the magnitude of the logFC, with lighter blue representing higher fold changes. Blue points indicate 6,521 significantly up-regulated (right) and down-regulated (left) genes, while black points represent non-significant genes, highlighting DEGs linked to oil composition differences

In the subsequent analysis, we screened 206 genes associated with oil biosynthesis (Table S6), based on established studies of fatty acid (FA), lipid, and triacylglycerol (TAG) biosynthesis pathways [9, 13, 18, 47, 48]. Heatmap analyses and volcano plot, presented in Fig. 4a and b, respectively, highlighted two distinct clusters of DEGs associated with oil biosynthesis, illustrating the differences in gene expression between high- and low-oleic genotypes.

Our comparative analysis of LO versus HO safflower genotypes identified seven key genes related to lipid biosynthesis (GO:0008610): Beta-carotene hydroxylase 2 (*BCH2*), very-long-chain aldehyde decarbonylase (*CER1*), Phosphatidylglycerophosphate phosphatase 1 (*PGPP1*), Monoacylglycerol lipase *ABHD6*, Lipid phosphate phosphatase epsilon 2 (*LPPE2*), and Phospholipase D zeta 1 (*PLDZ1*). CER1 is essential for synthesizing very long-chain FAs, while *ABHD6* plays a role in both lipid synthesis and degradation. In the FA biosynthesis pathway (GO:0006633), we identified 16 DEGs. Among these, five DEGs were associated with 3-oxoacyl-[acyl-carrier-protein (ACP)] synthase I and II (*KACS1* and KACS2). *KACS2* was up-regulated, whereas *KACS1* was down-regulated in the HO cultivar. *KACS* enzymes are essential for the initiation of fatty acid synthesis. Additionally, five genes encoding 3-ketoacyl-CoA synthase (*KCS*) were up-regulated in the HO cultivar. *KCS* is a key component in the FA elongase complex, important for synthesizing very long-chain fatty acids. Three genes involved in triglyceride biosynthesis (GO:0019432) were down-regulated: diacylglycerol O-acyltransferase 1 and 2 (*DGAT1* and *DGAT2*), and lysophospholipid acyltransferase 1 (*MBOA1*). *DGAT1* and *DGAT2* are critical for triglyceride synthesis, with *DGAT1* being a rate-limiting enzyme and *DGAT2* facilitating the final step of triglyceride formation. We also identified two novel *DGAT2* enzymes (MSTRG.17471.2_c0_g1 and MSTRG.8478.2_c0_g1) not mapped to the reference genome. These enzymes play vital roles in lipid metabolism. Among the 35 genes related to lipid metabolism (GO:0006629), we found a range of enzymatic activities involved in lipid synthesis, modification, and breakdown. Key enzymes identified include Δ-FA desaturases (*FAD2*, *FAD3*), phospholipases (*PLCD2*, *PLCD6*), acyltransferases (*WAXS1*, *WAXS5*), and various other lipid-modifying enzymes. Among the differentially expressed genes, phosphopantetheine adenylyltransferase (putatively *COAD*) and several lipases were notably down-regulated, while epoxide hydrolase 4 (*EPHX4*) and palmitoyl-monogalactosyldiacylglycerol Δ7-desaturase (*ADS3*) were up-regulated. In the FA metabolic process (GO:0006631), we identified 14 DEGs encoding critical enzymes such as 2-succinylbenzoate-CoA ligase (*MENE*), long-chain acyl-CoA synthetase 7 (*LACS7*), and acetyl-CoA synthetase (ACS). Gene MSTRG.9696.4_c0_g1, encoding *HAOX1*, was highly up-regulated (17.17 times) in the HO genotype. In the FA elongation process (GO:0030497), we observed differential expression of two genes: one encoding the 3-oxoacyl-[acyl-carrier-protein] reductase (*FABG*), which was down-regulated, and another encoding the (2 S)-[(R)-hydroxy(phenyl)methyl] succinyl-CoA dehydrogenase, which was up-regulated. These genes are involved in FA elongation and dehydrogenase functions.

**Fig. 5:**
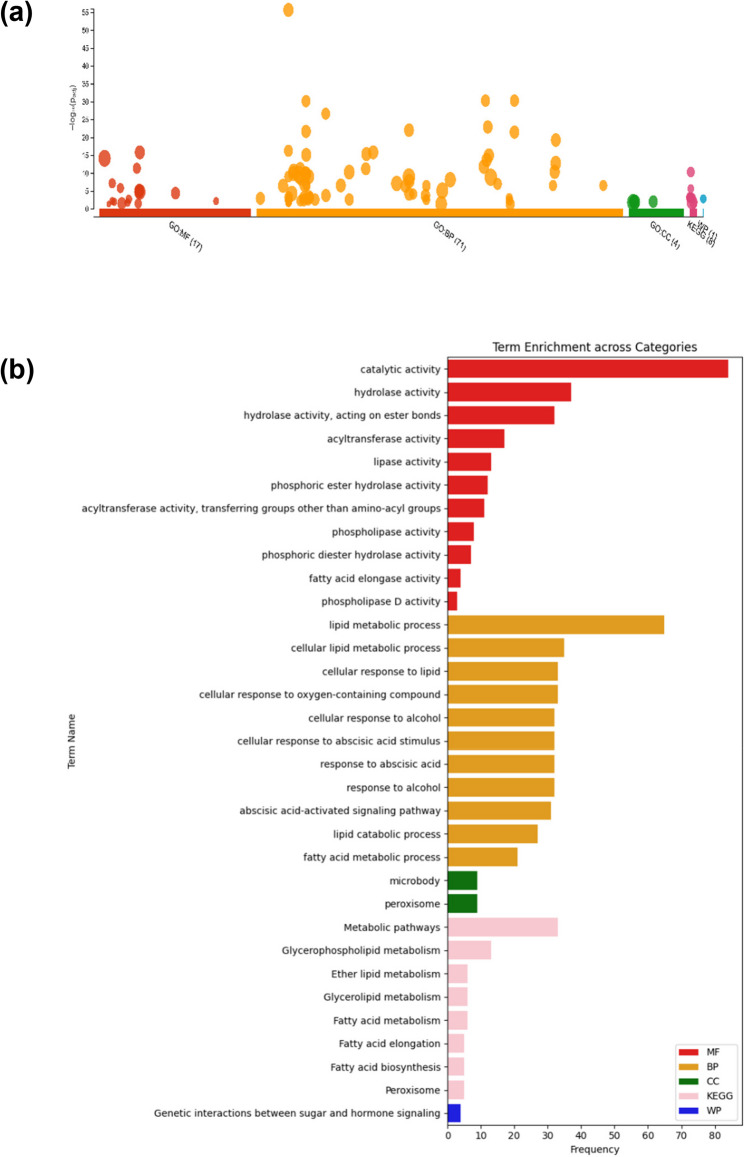
Functional profiling and term enrichment analysis. **(a**) Functional profiling using *Arabidopsis thaliana* as the reference species. The x-axis categorizes terms by Molecular Function (*MF*), Biological Process (*BP*), Cellular Component (*CC*), KEGG pathways, and WikiPathways (*WP*), while the y-axis shows the -Log10(padj). The size of the dots represents the number of associated genes for each term. **(b)** Bar plot displaying term enrichment across different categories. The frequency of enriched biological terms is indicated, with different colors representing the categories: MF (red), BP (orange), CC (green), KEGG (pink), and WP (blue)

Additionally, KEGG analysis identified 140 hits corresponding to *Arabidopsis thaliana* gene IDs. Functional profiling using the gProfiler web server showed that 71 genes were associated with biological processes (BP), 17 with molecular functions (MF), 8 with KEGG pathways, 4 with cellular components (CC), and 1 with WikiPathways (WP) (Fig. 5a). The most frequently occurring terms were “Catalytic activity” in the MF category, “Lipid metabolic processes” in the BP category, “Cytoplasm” in the CC category, and “Metabolic pathways” in the KEGG pathways category (Fig. 5b).

### Validation of gene expression profiles using qRT-PCR

**Fig. 6:**
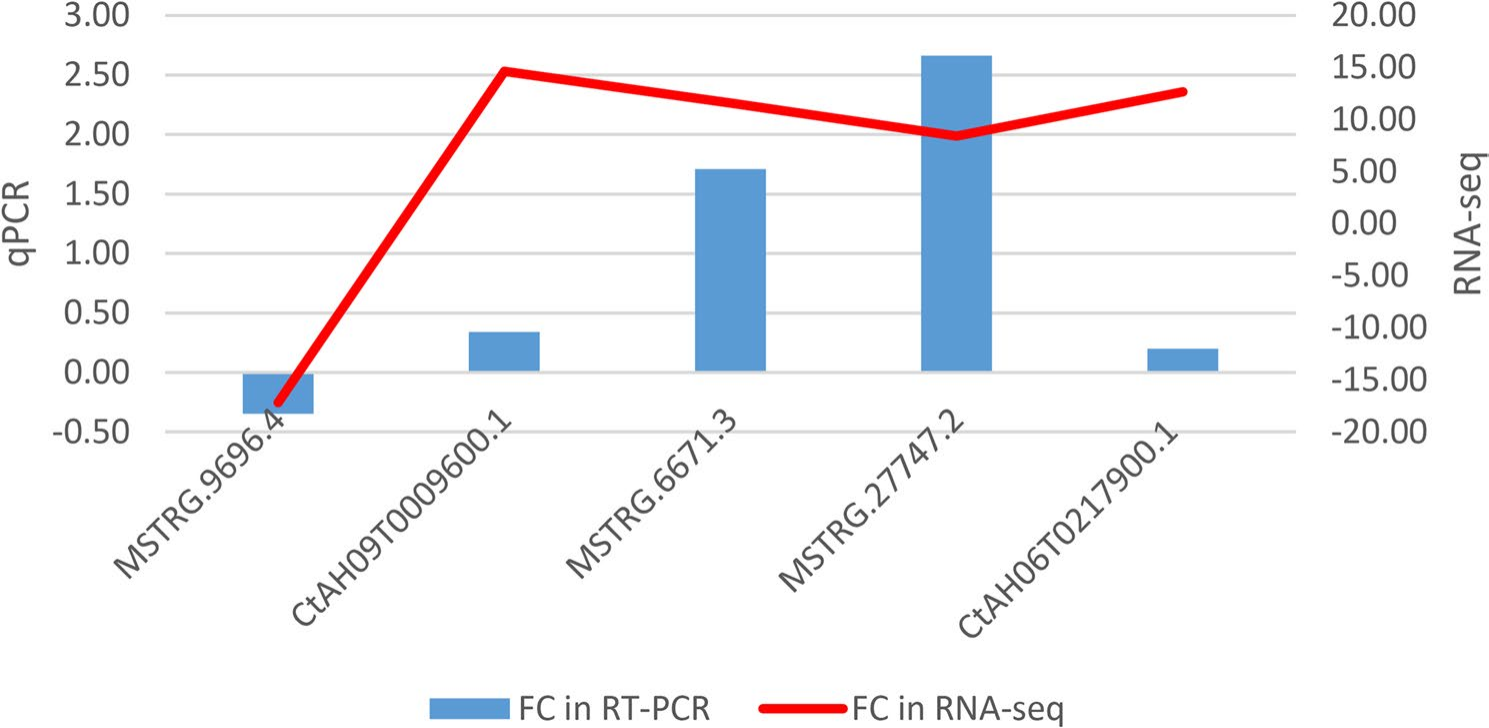
RT-qPCR validation of selected genes involved in lipid biosynthesis based on RNA-Seq analysis. Blue bars represent LogFC values obtained from RT-qPCR, while red lines indicate LogFC values from RNA-Seq. Genes analyzed include *Peroxisomal (S)−2-hydroxy acid oxidase GLO4* (*MSTRG.9696.4*), *Midasin (MDN1)* (*CtAH09T0009600.1*), *Enoyl-[acyl-carrier-protein] reductase (MECR)* (*MSTRG.6671.3*), *Delta (12)-fatty-acid desaturase (FAD2)* (*MSTRG.27747.2*), and Auxin response factor 18 (*ARF*) (*CtAH06T0217900.1*). Error bars represent standard error from three biological replicates. The results show a general consistency between RT-qPCR and RNA-Seq. RT-qPCR values were calculated using the ΔΔCt method (ΔΔCt = ΔCt(LO) – ΔCt (HO)), with HO as the calibrator; thus, positive LogFC values indicate higher expression in LO, while negative values indicate higher expression in HO

Five genes were selected based on different criteria for validating RNA-Seq data by qRT-PCR analyses in an additional experiment (Fig. 6). Two genes were chosen for their involvement in phytohormone signaling pathways: *Midasin (MDN1)* (*CtAH09T0009600.1*), which is associated with abscisic acid (ABA), and *Auxin Response Factor (ARF)* (*CtAH06T0217900.1*). The signalling of phytohormones has previously been reported to play a critical role in lipid metabolism and fatty acid biosynthesis [9, 49]. The remaining three candidate genes were selected due to their known functional roles in lipid biosynthesis: *Peroxisomal (S)−2-hydroxy acid oxidase GLO4* (*MSTRG.9696.4*), *Enoyl-[acyl-carrier-protein] reductase (MECR)* (*MSTRG.6671.3*), and *Delta (12)-fatty-acid desaturase (FAD2)* (*MSTRG.27747.2*). The relative expression levels obtained through qRT-PCR were consistent with the data from RNA-Seq between the two cultivars, HO (high oleic acid content) and LO (low oleic acid content).

## Discussion

In this study, we combined GWAS and RNA-seq to explore the genetic basis of oil and FA biosynthesis in safflower. Our findings highlight the critical role of seed yield in determining oil yield, with significant correlations between FAs revealing interconnected biosynthetic pathways.

We observed a strong positive correlation between seed yield and oil yield (*r* = 0.94^***^), underscoring the obvious role of seed production in determining overall oil output. This finding aligns with previous research demonstrating that increasing seed yield is a key factor in enhancing oil yield. For instance, similar correlations have been reported in oilseed crops like soybean, where seed yield was shown to be the primary driver of oil yield [50]. This suggests that breeding strategies focusing on improving seed yield could be highly effective for boosting oil production. In contrast, the moderate correlation between oil content and oil yield (*r* = 0.35^***^) indicates that while oil content contributes to oil yield, its impact is secondary to seed yield. This observation is consistent with findings in other oilseed crops, where oil content alone does not fully explain variations in oil yield [51]. Therefore, while optimizing oil content is important, prioritizing seed yield remains crucial for improving overall oil yield.

Our analysis of FA composition revealed a significant positive correlation between MA and PA (*r* = 0.55***), suggesting that these fatty acids are synthesized through interconnected pathways. The correlation between SA and PA (*r* = 0.26***) further implies that SA may be synthesized from PA through elongation processes, which is consistent with known metabolic pathways [18].

Additionally, the positive relationship between OA and EA (*r* = 0.25**) suggests that OA could serve as a precursor for EA through elongation and desaturation. This finding is supported by previous research highlighting the role of OA in the synthesis of longer-chain FAs such as EA [52, 53].

GWAS identified 64 significant SNP loci associated with oil traits, highlighting the complex genetic basis underlying oil composition in safflower. This study provides crucial insights into the genetic factors that influence oil production, highlighting the roles of specific chromosomes and candidate genes. Chromosomes 5 (at 85,633,330 bp) and 8 (at 3,135,949 bp) were particularly notable for their associations with oleic and linoleic acids. On chromosome 5, we identified 297 candidate genes, including *KASC2*, which was up-regulated in HO genotypes based on RNA-seq data. KASC2’s up-regulation in HO genotypes suggests its crucial involvement in FA biosynthesis, potentially influencing oleic acid content and making it a promising target for genetic improvement. A previous study has shown that similar genes are involved in the regulation of FA synthesis, emphasizing their importance in breeding programs aimed at enhancing oil quality [10].

Transcription factors (TFs) from the *ARF* (Auxin Response Factor) family, associated with SNP loci on chromosome 5, are implicated in the regulatory networks governing FA biosynthesis. ARFs are known to modulate gene expression in response to auxin, a plant hormone that influences various developmental processes, including lipid metabolism [49].

Chromosome 10 also demonstrated significant enrichment of molecular trait associations (MTAs) related to oil traits, highlighting the presence of important genes involved in oil synthesis and accumulation. Notably, candidate genes such as *GDL79*, *GDL67*, and Oleosin Cor a 13 (*OLE13*) were identified. These genes play critical roles in oil body formation and stabilization, which are essential for oil storage and quality. Oleosins, for instance, are known to be integral components of oil bodies, contributing to the stability of stored lipids [54, 55].

We identified 6,521 DEGs through transcriptome analysis of safflower seeds. The candidate genes detected, known to regulate oil and FA content, include the well-documented *FAE1* (3-Oxoacyl-[acyl-carrier-protein (ACP)] synthase III), *FATB*, *FAD2*, *FAD3* (Fatty Acid Desaturase 3), Ketoacyl-ACP Synthase II (*KAS II*), Ketoacyl-ACP Synthase I (*KAS I*), and DGAT genes. Recent analyses in oilseed crops including Camelina and others have shown that *FAD2* and *FAD3* gene families are major determinants of unsaturated fatty acid composition [56, 57], and that *DGAT* plays a central role in triacylglycerol assembly driving fatty acid composition and oil content [58]. In the present study, *FAD2* and *FAD3* were up-regulated in the LO cultivar and down-regulated in the HO cultivar. The down-regulation of these genes in the HO cultivar suggests a decrease in the conversion rate of oleic acid, which corresponds with the observed increase in oleic acid content in HO genotypes [9]. This finding is consistent with research demonstrating that reduced activity of *FAD2* and *FAD3* in high-oleic plants leads to higher levels of oleic acid accumulation [48]. Moreover, *FAD3* is responsible for converting precursor fatty acids, such as linoleic acid (LA), into α-linolenic acid (ALA), a key omega-3 polyunsaturated fatty acid (PUFA) [59]. The modulation of these biosynthetic pathways reflects the genetic and molecular adjustments that contribute to the FA composition observed in safflower genotypes. The up-regulation of *SAD* in HO genotypes indicates an enhanced production of oleic acid. The up-regulation of *SAD* in HO genotypes indicates an enhanced production of linoleic acid. *SAD* is crucial for the conversion of stearic acid to oleic acid, and its increased expression in HO genotypes suggests a shift in the FA profile towards higher linoleic acid content due to changes in the pathway that subsequently affect linoleic acid production [60]. Previous research has demonstrated that *SAD* activity influences overall FA composition and can indirectly impact linoleic acid levels through complex metabolic interactions [60, 61]. The up-regulation of *SAD* in our high-linoleic genotypes supports the notion that this enzyme plays a significant role in modulating FA profiles, contributing to the observed increase in linoleic acid.

Additionally, our study identified 110 TFs and 55 DEGs involved in the ABA (abscisic acid) signaling pathway (Table S7). This pathway has previously been demonstrated to play a critical role in lipid metabolism and the biosynthesis of FAs [9]. Among the transcription factors, *MYB4*, *LIMYB*, and *AP21* emerged as noteworthy candidates that were consistently identified through both GWAS and RNA-seq analyses. *MYB4* has been shown to influence FA composition and stress tolerance in plants [62].

Overall, integrating GWAS and RNA-seq data has provided a comprehensive view of the genetic and molecular factors influencing oil and FA biosynthesis in safflower. The significant SNPs and candidate genes identified offer valuable markers for genetic improvement. These findings can be used to develop safflower varieties with enhanced oil content and tailored FA profiles. Additionally, the discovery of novel genes and TFs presents opportunities for targeted genetic manipulation to optimize oil yield and quality.

## Conclusions

This study successfully integrated GWAS and RNA-seq analyses to identify key genomic regions and genes regulating oil content and fatty acid biosynthesis in safflower. A total of 64 significant loci were detected across the genome, with chromosome 10 emerging as a major hotspot for oil-related traits. Candidate genes such as *KASC2*,* FAD2*,* FAD3*,* SAD*,* DGAT1*,* DGAT2*, and *OLE13* were identified as critical regulators of lipid metabolism and oil accumulation. We identified several TFs in our study, among which *MYB4*,* LIMYB*, and *AP21* emerged as noteworthy candidates consistently detected through both GWAS and RNA-seq analyses, suggesting their central regulatory roles in fatty acid biosynthesis. In addition, our analysis revealed multiple TFs and DEGs associated with the ABA signaling pathway, which is known to play a crucial role in lipid metabolism and fatty acid biosynthesis. The differential expression of these genes between high- and low-oleic lines confirms their functional importance in determining fatty acid composition.

Overall, the integration of association mapping with transcriptomic data provides a comprehensive framework linking genetic variation to metabolic function and offers valuable molecular targets for safflower breeding programs aimed at improving oil yield, composition, and overall quality.

## Supplementary Information

Supplementary Material 1

Supplementary Material 2

Supplementary Material 3

## Data Availability

All data generated or analyzed during this study are included in this article and its supplementary information files. The transcriptome and GBS sequence data have been deposited in GenBank under BioProject accession numbers PRJNA865057, SUB15872891, and PRJNA1338490.
