# Supplementary material for: Integrative GWAS and transcriptomic analyses identify candidate genes associated with oil content and fatty acid composition in safflower
Source: BMC Plant Biol. 2026 Jan 29;26:372. doi: 10.1186/s12870-025-08024-1 (PMC12930970; doi:10.1186/s12870-025-08024-1)

**Supplemental Figure**

Fig. S1 Circular Manhattan plots display SNPs significantly associated with traits. The plots feature concentric circles representing the association analyses conducted using the mixed linear model (MLM), general linear model (GLM), and fixed and random effects model for circulating probability unification (FarmCPU) methods. Each dot corresponds to an SNP, with its radial distance from the center reflecting its p-value. Red dotted lines indicate the Bonferroni correction threshold at p < 0.05. The outermost heatmap ring shows SNP density across chromosomes, with colors ranging from green (low density) to red (high density). Chromosomes 1 to 12 are labeled around the outer circle. The p-values in −log10 scale are represented by black vertical lines. The chart on the right shows quantile-quantile (QQ) plots of observed versus expected p-values based on the MLM, GLM, and FarmCPU methods.


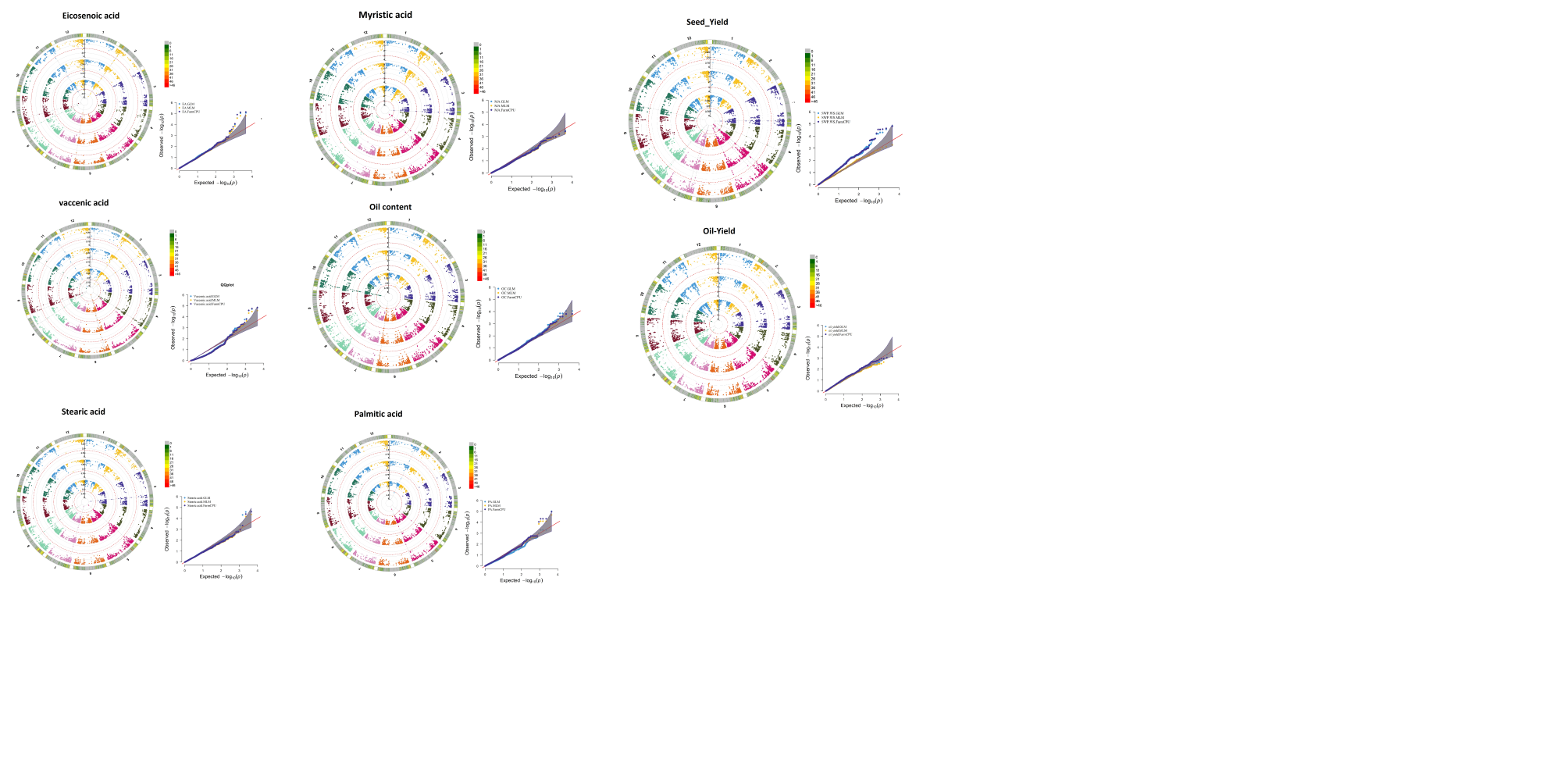

Supplement: Supplementary file 1 — Supplementary Material 1 [file 12870_2025_8024_MOESM1_ESM.docx]
