# Supplementary material for: Integrative GWAS and transcriptomic analyses identify candidate genes associated with oil content and fatty acid composition in safflower
Source: BMC Plant Biol. 2026 Jan 29;26:372. doi: 10.1186/s12870-025-08024-1 (PMC12930970; doi:10.1186/s12870-025-08024-1)
Supplement: Supplementary file 2 — Supplementary Material 2 [file 12870_2025_8024_MOESM2_ESM.docx]

**Author Information:**

**Somayeh Sardouei-Nasab***

Leibniz Institute of Plant Genetics and Crop Plant Research (IPK), Gatersleben, Germany

Current address: Research and Technology Institute of Plant Production, Afzalipour Research Institute, Shahid Bahonar University of Kerman, Kerman, Iran
 **Email:** [sardoueinasab@uk.ac.ir](mailto:sardoueinasab@uk.ac.ir)

**Fatemeh Mahdavinasab**
 Department of Plant Production and Genetics, College of Agriculture, Shahid Bahonar University of Kerman, Kerman, Iran
 **Email:** [fatemeh.mahdavii1378@gmail.com](mailto:fatemeh.mahdavii1378@gmail.com)

**Ghasem Mohammadi-Nejad**
Research and Technology Institute of Plant Production, Afzalipour Research Institute, Shahid Bahonar University of Kerman, Kerman, Iran
 Email: [mohammadinejad@uk.ac.ir](mailto:mohammadinejad@uk.ac.ir)

**Reza Haghi**

Leibniz Institute of Plant Genetics and Crop Plant Research (IPK), Gatersleben, Germany
 Email: [haghi@ipk-gatersleben.de](mailto:haghi@ipk-gatersleben.de)

**Azam Nikbakht-Dehkordi**
Research and Technology Institute of Plant Production, Afzalipour Research Institute, Shahid Bahonar University of Kerman, Kerman, Iran
Email: [azam.nikbakht.dehkordi@gmail.com](mailto:azam.nikbakht.dehkordi@gmail.com)

**Zahra Nemati***

Leibniz Institute of Plant Genetics and Crop Plant Research (IPK), Gatersleben, Germany
Email: [nemati@ipk-gatersleben.de](mailto:nemati@ipk-gatersleben.de)
